# Thymic carcinoma recurring 11 years post-extended surgery: a case report

**DOI:** 10.1186/s44215-025-00190-w

**Published:** 2025-02-26

**Authors:** Kiyoki Okamoto, Takashi Kanou, Sachi Kawagishi, Hideki Nagata, Eiichi Morii, Yasushi Shintani

**Affiliations:** 1 Department of General Thoracic Surgery, Osaka University Graduate School of Medicine, 2-2 Yamadaoka, Suita, Osaka 565-0871 Japan; 2 Department of Pathology, Osaka University Graduate School of Medicine, Osaka, Japan

**Keywords:** Thymic carcinoma, Long-term recurrence, Aorta

## Abstract

**Background:**

Thymic carcinoma is a rare type of tumor originating in the thymus, making up about 15–20% of all thymic epithelial tumors. It typically has a poor prognosis, especially in advanced stages, with low 5-year survival rates. Cases where the cancer recurs more than 10 years after surgery are extremely uncommon. Additionally, there are very few reports about the outcomes of patients who undergo aortic resection as part of their treatment for thymic carcinoma.

**Case presentation:**

A 68-year-old male was diagnosed with thymic squamous cell carcinoma classified as Masaoka stage III following the detection of an anterior mediastinal mass during a routine health examination. The patient underwent preoperative treatment, which included two cycles of chemotherapy (cisplatin and docetaxel) and 60 Gy of mediastinal radiotherapy, followed by an extensive surgical procedure comprising extended thymectomy, resection of the ascending aorta and superior vena cava, and wedge resection of the right upper lobe. Postoperative pathological examination revealed ypT3N0M0 disease, corresponding to ypStage IIIa disease, and the patient remained disease-free for 10 years. However, at 11 years after surgery, imaging revealed new nodules in the left lung. Surgical resection confirmed these nodules as metastatic lesions originating from the thymic carcinoma.

**Conclusions:**

This case highlights the critical need for long-term monitoring of thymic carcinoma patients, extending beyond the standard 5-year follow-up due to the potential for late recurrence, even in initially disease-free patients. Furthermore, our findings indicate that aortic resection, when carefully selected, can contribute to favorable long-term outcomes in advanced cases. This report enhances the limited literature on the long-term prognosis of thymic carcinoma, particularly following major vascular resection, and underscores the importance of a multidisciplinary approach to optimize patient management and improve outcomes.

## Introduction

Thymic carcinoma is an uncommon type of thymic epithelial tumor, accounting for approximately 10–15% of all thymic tumors [1]. Patients diagnosed with advanced stages of this cancer face a challenging prognosis, and it is known as a disease with a poor prognosis. Complete resection is a prognostic factor for thymic carcinoma [2–4]; however, recurrence can still occur even after complete resection. Reports of initial recurrence occurring more than 10 years postoperatively are extremely rare [5]. Herein, we report a case of thymic carcinoma that recurred more than 10 years after complete resection through trimodal treatment including extensive surgery which we previously reported [6].

## Case presentation

**Fig. 1:**
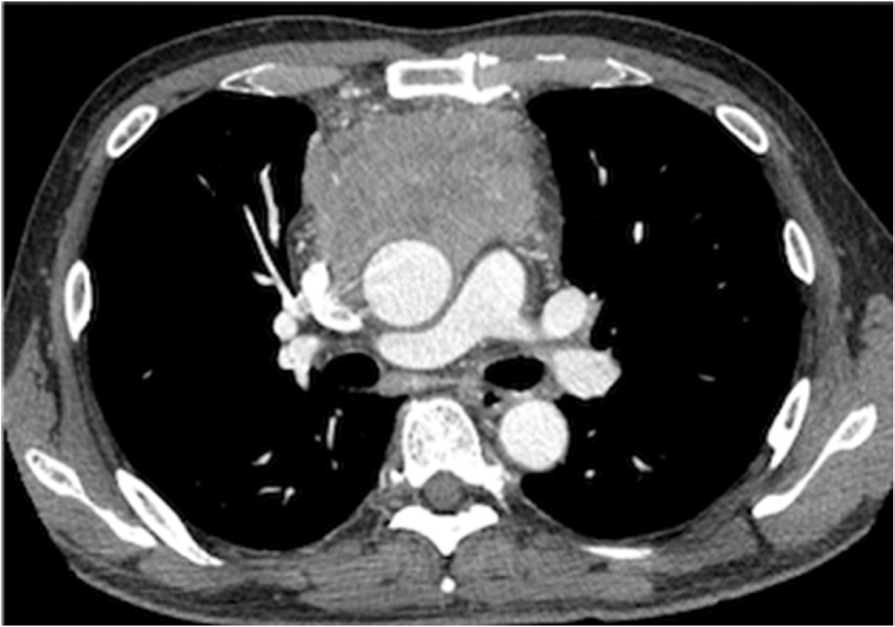
Initial contrast-enhanced chest CT image showing an irregular 8.4 × 6.0 cm mass in the anterior mediastinum. The mass abuts the ascending aorta and compresses the SVC, suggesting invasion

**Fig. 2:**
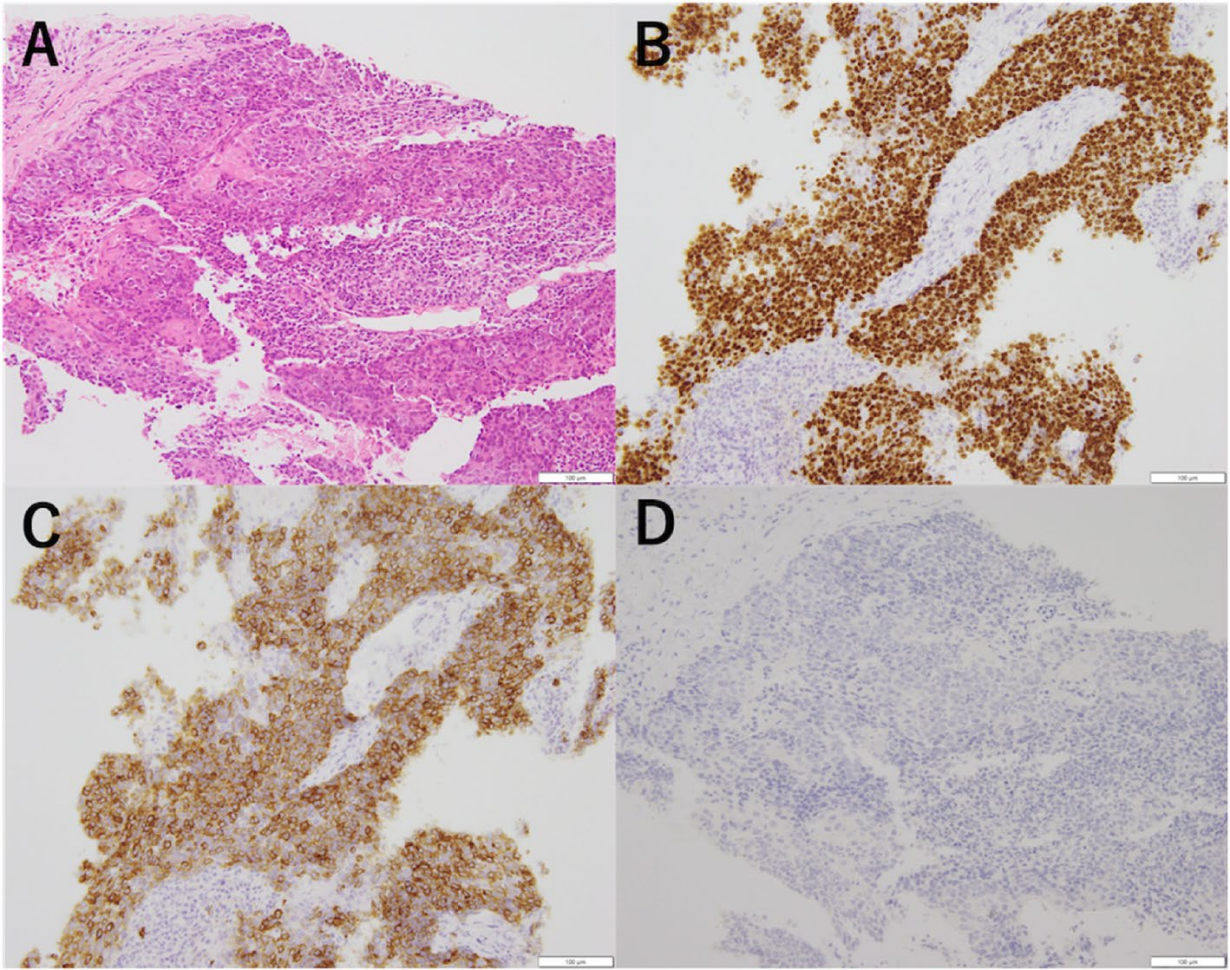
CT-guided needle biopsy was performed for diagnostic purposes. Pathological findings revealed tumor cells with prominent nucleoli and eosinophilic cytoplasm arranged in a cobblestone pattern, with areas of keratinization (**A**). Immunohistochemistry demonstrated p63 positive (**B**), CD117 (c-kit) positive (**C**), and TdT negative (**D**), supporting a diagnosis of thymic squamous cell carcinoma

**Fig. 3:**
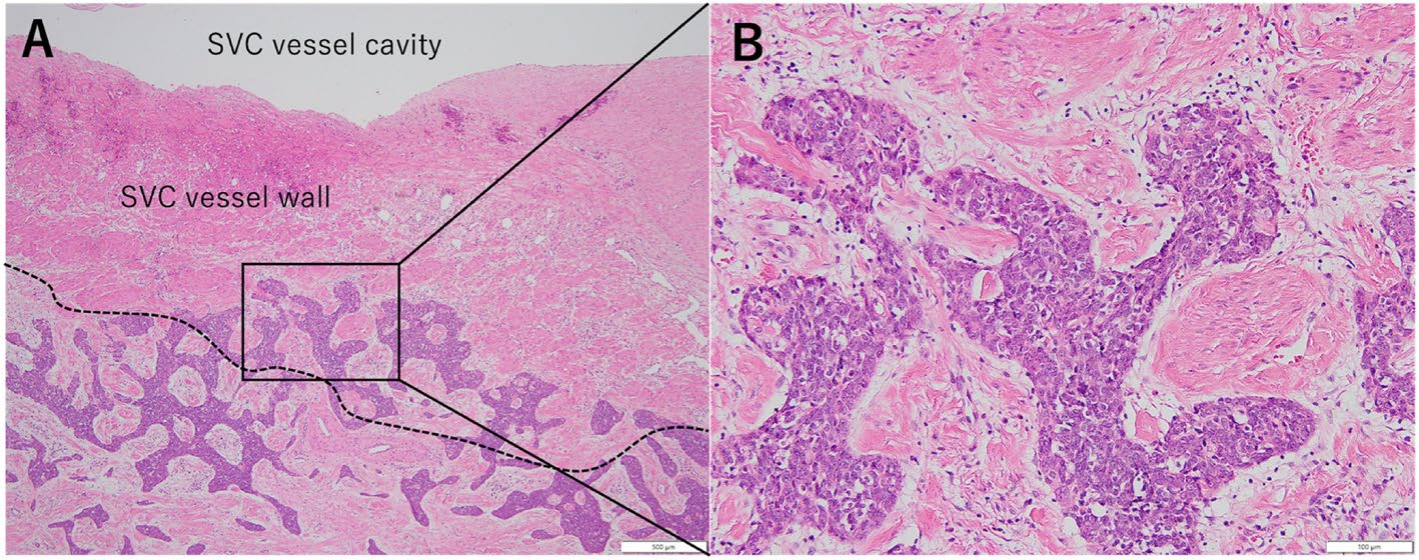
Initial postoperative pathological findings showing typical cells with coarse chromatin and prominent nucleoli proliferating in nests with invasion into the SVC wall (**A**, **B**)

A 68-year-old male who presented to our hospital after a routine health check revealed an abnormal shadow on his chest radiograph. Contrast-enhanced chest computed tomography (CT) revealed an anterior mediastinal mass (Fig. 1). A CT-guided needle biopsy was performed via a vertical approach through the left parasternal space and confirmed the diagnosis of thymic squamous cell carcinoma which was classified as Masaoka stage III (Fig. 2). As part of the preoperative treatment, the patient underwent two cycles of chemotherapy with cisplatin and docetaxel, along with 60 Gy of mediastinal radiotherapy. Following this treatment, we performed extended thymectomy combined with resection of the ascending aorta and superior vena cava (SVC), wedge resection of the right upper lobe, replacement of the ascending aorta, and SVC reconstruction [6]. Permanent pathological examination confirmed the diagnosis of squamous cell carcinoma with tumor invasion into the SVC wall and adjacent tumor cells to the aortic wall (Fig. 3). No malignancy was detected in the regional lymph nodes or pericardium. Based on these findings, the tumor was staged as ypT3N0M0, ypStage IIIa according to the American Joint Committee on Cancer 8th Edition criteria, which is consistent with Masaoka stage III.

**Fig. 4:**
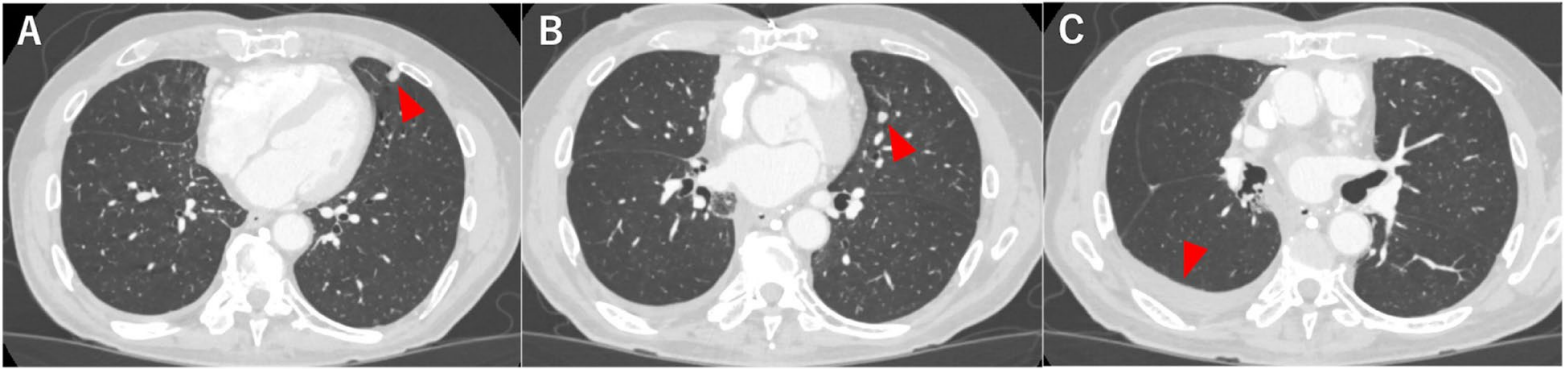
Contrast-enhanced chest CT image taken before the second surgery showing two solid nodules in the left lung lingular segment measuring 8 × 7 mm (**A**) and 7 × 5 mm (**B**), with a small amount of right pleural effusion (**C**)

**Fig. 5:**
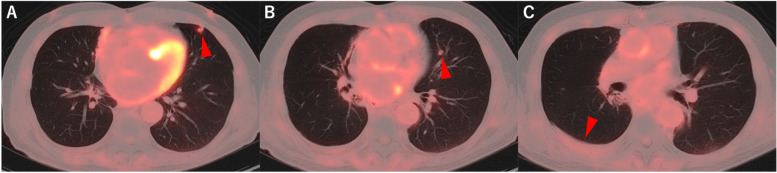
PET-CT at recurrence revealed an SUVmax of 3.4 for two small nodules in the left lung lingular segment (**A**, **B**). A small amount of right pleural effusion without abnormal uptake was also observed (**C**)

The patient was monitored with contrast-enhanced CT every 6 months until the fifth postoperative year, and annually thereafter and remained disease-free for 10 years postoperatively. However, in the 11th postoperative year, plain chest CT conducted at his local clinic revealed increased right pleural effusion and multiple nodules in the left lung. The patient was subsequently referred to our department for further investigation and management. Upon admission, all hematological and biochemical parameters were within normal limits. The levels of tumor markers, including CEA (2.0 ng/mL), CYFRA (0.7 ng/mL), and ProGRP (35.1 pg/mL), were also within normal ranges. Contrast-enhanced chest CT revealed two solid nodules in the lingular segment of the left lung (Fig. 4), whereas positron emission tomography (PET)-CT demonstrated fluorodeoxyglucose uptake with a maximum standardized uptake value (SUVmax) of 3.4 in these nodules. The right pleural effusion showed no abnormal uptake (Fig. 5), and contrast-enhanced brain CT revealed no evidence of brain metastasis. On the basis of these findings, metastasis from thymic carcinoma was highly suspected as the primary diagnosis, although differential considerations included secondary pulmonary metastasis from another organ’s primary cancer, primary lung cancer, and inflammatory nodules. After these potential diagnoses were evaluated, since the right pleural effusion was minimal and a sufficient amount of tissue was deemed necessary for a detailed diagnosis, we proceeded with diagnostic resection of the lung lesions. Given the previous right lung resection and anticipated severe adhesions, we initially opted to perform resection on the left lung nodules. The surgery was conducted under general anesthesia via a completely thoracoscopic approach. After extensive adhesiolysis within the thoracic cavity, only one of the two tumors in the lingular segment of the left lung was palpated and resected using an automatic stapler. The operation lasted 65 min with minimal blood loss. The patient’s postoperative recovery was uneventful, and he was discharged home on postoperative day 8.

**Fig. 6:**
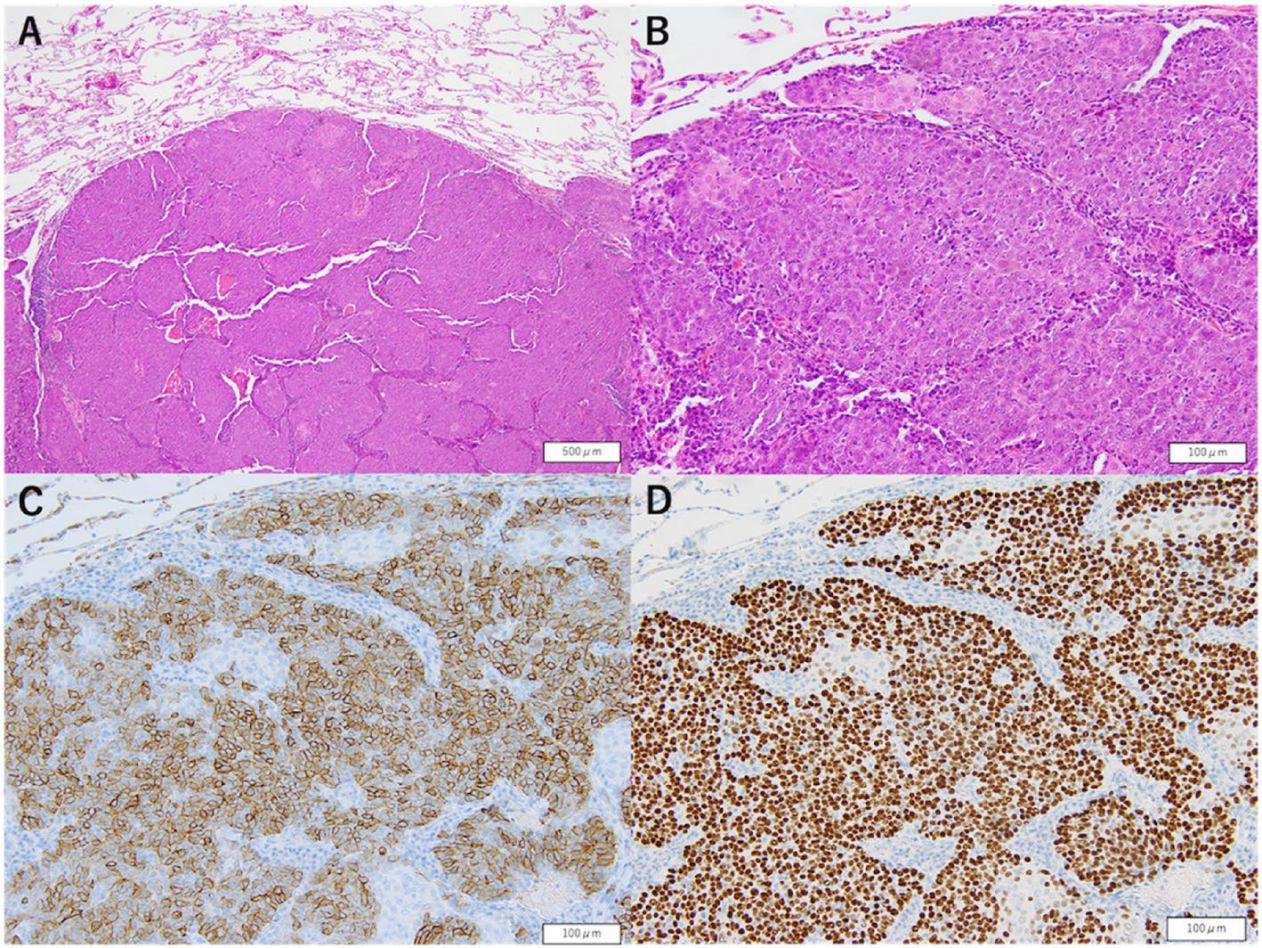
Pathological findings at the time of the second surgery via hematoxylin and eosin staining demonstrating that tumor cells with nuclear atypia proliferate in nests and are distinctly demarcated from the surrounding normal lung tissue (**A**, **B**) Immunohistochemical staining revealed positive results for CD117 (c-kit) (**C**) and p40 (**D**), which aligned with the characteristics of known thymic carcinoma metastases

Histopathological examination of the resected specimen revealed findings consistent with metastatic thymic carcinoma (Fig. 6). The nodule in the left lung lingular segment was thus confirmed as a metastatic lesion from thymic carcinoma. Given the clinical context, another nodule in the lingular segment of the left lung and right pleural effusion was also regarded as metastases of thymic carcinoma. A multidisciplinary team conference recommended the initiation of systemic chemotherapy, and the patient is currently receiving chemotherapy (carboplatin + paclitaxel) at his primary care facility. If disease progression is observed, lenvatinib should be considered the next line of therapy.

## Discussion

Thymic carcinomas are often large and infiltrate surrounding organs at the time of discovery. Consequently, many cases are in advanced stages (Masaoka stage III or IV) at diagnosis, with recent reports suggesting an overall 5-year survival rate of 36–61.1%, making it a disease with a poor prognosis [2, 7, 8]. The complete resection of thymic carcinoma has improved the 5-year survival rate to 58–85.1%, confirming the importance of surgical intervention [9]. Additionally, preoperative and postoperative chemotherapy and radiotherapy have been shown to improve survival, emphasizing the importance of multidisciplinary treatment, which includes surgery, chemotherapy, and radiotherapy [10].

In this case, long-term survival was achieved after complete resection with combined resection of the aortic arch and superior vena cava for advanced-stage thymic carcinoma (Masaoka stage III) following preoperative chemoradiotherapy, with distant metastasis occurring 11 years postoperatively. Yau-Lin et al. reported that tumor infiltration of the superior vena cava, pulmonary artery, and aorta are poor prognostic factors after surgery for thymic carcinoma [11]. However, there have been few reports on long-term outcomes after thymic carcinoma resection with aortic resection. In our review of the literature, there are only five reported cases regarding the prognosis after thymic cancer surgery involving concomitant resection of the aorta, and most studies provide only short-term disease-free survival data (Table 1) [12–15]. We present six cases from our institution including cases which we previously reported (Table 2) [16, 17]. Among these patients, the average age was 60 years (range,45–74; median, 60), with 4 males and 2 females. Clinically, 3 patients had Masaoka stage III disease, and 3 had stage IV disease; all patients underwent surgery and R0 resection was achieved in all patients. The postoperative observation period ranged from 4 months to 11 years and 5 months, with 3 patients experiencing recurrence and no deaths during the observation period. Although aortic resection during thymic carcinoma surgery is often avoided because of the high complication and surgical mortality rates, long-term survival beyond 5 years was achieved in five cases, including two cases exceeding 10 years, suggesting that aortic resection can be a viable option when carefully considered. No fatal postoperative complications were observed in our patients, indicating that case selection was appropriate.

**Table 1:** Previously reported cases of thymic carcinoma with combined aortic resection

Author	pMasaoka stage	pTNM	pStage	Combined resected organs	Resection	Recurrence	Outcome	Preoperative therapy	Postoperative therapy
Oyama [12]	III	unknown	unknown	Ao arch wedge, Lt. S3	R0	None	11 m	CDDP + BLM + VLB, RT	None
Takahashi [13]	III	pT4N0M0	IIIb	Ao arch, BCA, Lt. CCA, Lt. SCA, Lt. BCV, LUL, Lt. VN	R0	13 m (anterior mediastinum)	Unknown	CP, RT	RT
Michael Ried 1 [14]	III	pT4N0M0	IIIb	Ao arch, main PA, Lt. BCV	R2	Increase in residual tumor	7 m dead	PAC	None
Michael Ried 2 [14]	III	pT4N0M0	IIIb	Ao arch, BCA, Lt. CCA, Lt. SCA, Lt. BCV, RUL wedge, LUL wedge	R0	None	8 m	PAC	None
Oka [15]	III	pT4N0M0	IIIb	Ao arch, BCA, Lt. CCA, Lt. SCA, Lt. BCV, LUL, Lt. VN	R0	None	3 y	none	None

*Ao*, aorta; *BCA*, brachiocephalic artery; *CCA*, common carotid artery; *SCA*, subclavian artery; *PA*, pulmonary artery; *BCV*, brachiocephalic vein; *RUL*, right upper lobe; *Rt*, right, *Lt*, left; *LUL*, left upper lobe; *VN*, vagus nerve; *m*, month; *y*, year; *CDDP*, cisplatin; *BLM*, bleomycine; *VLB*,vinblastine; *RT*, radiation therapy; *CP*, carboplatin + paclitaxel; *PAC*, cisplatin + doxorubicin + cyclophosphamid

**Table 2:** Summary of thymic carcinoma cases undergoing combined aortic resection at our institution

Case	Age	Sex	Histological type	cMasaoka stage	cTNM	Preoperative therapy	Combined Resected Organs	ypTNM	ypStage	Postoperative therapy	Recurrence	Recurrence therapy	Outcome
1	57	M	Sq	IVb	T4N2M0	CDDP + DTX, RT	Ao arch, SVC, RUL wedge	T4N0M0	IIIb	none	11 y 2 m (Lt. lung)	CP	11 y 5 m
2	65	F	Sq	III	T4N0M0	CP, RT	Ao arch, Rt. VN, Rt. PN	T1bN0M0	I	none	3 y 0 m (Lt. thyroid gland)	CP (PD) → Lenvatinib^a^ (SD)	10 y 7 m
3 [16]	74	M	Sq	III	T4N0M0	ADOC → CP	Ao arch、Lt. VN, Lt. RLN, Lt. PN	T3N0M0	IIIa	none	None	none	7 y
4 [17]	45	M	Sq	IVa	T4N0M1a	CP → TGO	Ao arch, main PA, Lt. BCV, Lt. Pneumonectomy, Lt. VN, Lt. PN	T2N0M0	II	none	3 y 1 m (paratracheal lymph nodes)	RT	6 y 9 m
5	61	M	Sq	III	T4N0M0	CP, RT	Ao arch, LUL, Lt. VN, Lt. PN, Lt. SNT	T4N0M1a	IVa	none	None	None	5 y 8 m
6	59	F	Sq	IVa	T4N0M1a	CDDP + ETP, RT	Ao arch, main PA, Lt. BCV, LUL	T3N0M0	IIIa	none	None	None	4 m

*M*, male; *F*, female; *Sq*, squamous cell carcinoma; *CDDP*, cisplatin; *DTX*, doxorubicin; *RT*, radiation therapy; *CP*, carboplatin + paclitaxel; *ADOC*, doxorubicin + cisplatin + vincristine + cyclophosphamide; *TGO*, tegafur + gimeracil + oteracil; *ETP*, etoposide; *Ao*, aorta; *SVC*, superior vena cava; *RUL*, right upper lobe; *VN*, vagus nerve; *PN*, phrenic nerve; *RLN*, reccurent laryngeal nerve; *PA*, pulmonary artery; *BCV*, brachiocephalic vein; *LUL*, left upper lobectomy; *SNT*, sympathetic nerve trunk; *Rt*, right; *Lt*, left; *m*, month; *y*, year

^a^Discontinued due to adverse effects

Hishida et al. studied long-term outcomes after surgery for thymic carcinoma and reported significant differences in overall survival and recurrence-free survival between stages I/II and III/IV at both 5 and 10 years postoperatively [3]. Previous reports suggest that the risk of disease progression between 5 and 10 years postoperatively is minimal, with Hamaji et al. reporting a median time to first recurrence of 10 months (range, 2–54 months) [18]. Accordingly, the current Japanese guidelines recommend a follow-up period of more than 5 years after surgery for thymic carcinoma. Retrospective studies have been the main source of reports on the long-term outcomes of thymic carcinoma, and only three cases have reported detailed reports on recurrence after 10 years (Table 3) [5, 19, 20]. In this case, recurrence occurred 11 years after the initial surgery, prompting reconsideration of the follow-up period for thymic carcinoma after surgery. In other malignancies, factors such as tumor size, age, and lymph node metastasis have been reported as predictors of late recurrence [21, 22]. No definitive predictive factors were identified within the cases of this study. However thymic NEC and MEC have been reported in the literature to recur more than 20 years after surgery. Moreover, the cellular origin of late relapse in these patients is believed to be disseminated tumor cells that become reactivated after a prolonged period of dormancy [23]. A similar concept may need to be considered in thymic carcinoma as well. Regarding the treatment of recurrence in cases from our institution (Table 2), Case 1 is currently under follow-up shortly after the initiation of chemotherapy. In Case 2, left thyroid metastasis was observed 3 years after surgery, and local control was achieved with radiotherapy. However, bilateral pulmonary metastases were identified 6 years and 6 months postoperatively. Chemotherapy was initiated but resulted in progressive disease. Following the initiation of lenvatinib therapy, the patient has achieved stable disease for seven years. In Case 4, paratracheal lymph node metastasis was observed 3 years and 1 month postoperatively, and local control was successfully achieved with radiotherapy. Considering these cases, systemic therapy with novel agents and local treatments for oligometastases may also be effective in managing postoperative recurrence of thymic carcinoma. These findings support the necessity of long-term follow-up for advanced thymic carcinoma.

**Table 3:** Case series of thymic carcinoma recurring more than 10 years after surgical resection

Author	Histological type	Preoperative therapy	Prosedure	Resection	pMasaoka stage	Postoperative therapy	Recurrence interval	Recurrence site	Recurrence therapy
Toyokawa [19]	NEC	unknown	Thymectomy	R1	I	None	24 y	Local reccurrence	None
Yabuki [5]	Sq	none	Thymectomy + LUL Wedge	R0	III	CDDP + ETP, RT	10 y	Lt. malignant pleural effusion	CP
Hayashi [20]	MEC	none	Extended thymenctomy + RUL Wedge	R0	III	None	21 y	Rt. pleural dissemination	None

*NEC*, neuroendocrine carcinoma; *Sq*, squamous cell carcinoma, *MEC*, mucoepidermoid carcinoma; *LUL*, left upper lobe; *RUL*, right upper lobe; *CDDP*, cisplatin; *ETP*, etoposide; *RT*, radiation therapy; *y*, year; *Lt*, left; *Rt*, right; *CP*, carboplatin + paclitaxel

## Conclusions

For advanced thymic carcinoma, follow-up for more than 10 years may be considered even after complete resection. Additionally, aggressive thymic carcinoma resection, including aortic resection, may achieve favorable long-term outcomes when combined with appropriate techniques and careful patient selection.

## Data Availability

Not applicable.

## Abbreviations

CT: Computed tomography
PET: Positron emission tomography
SUVmax: Maximum standardized uptake value
SVC: Superior vena cava
